# Self-Service System for the Family Members of ICU Patients: A Pilot Study

**DOI:** 10.3390/healthcare10030467

**Published:** 2022-03-02

**Authors:** I-Chiu Chang, Ying-Hui Hou, Li-Jung Lu, Yu-Chen Tung

**Affiliations:** 1 Department of Information Management, National Chung Cheng University, Chiayi 62102, Taiwan; misicc@mis.ccu.edu.tw (I.-C.C.); y0058@yuanhosp.com.tw (L.-J.L.); 2 Department of Health Industry Management, Kainan University, Taoyuan 33857, Taiwan; yhhou@mail.knu.edu.tw; 3 Department of Nursing, Yuan’s General Hospital, Kaohsiung 802635, Taiwan; 4 Department of Nursing, Chi Mei Medical Center, Tainan 71004, Taiwan

**Keywords:** intensive care unit, family member requirements, informational systems, self-service technology, importance–performance analysis

## Abstract

Family members of intensive care unit patients are often experience high anxiety and require more information about the patients. However, most Taiwanese healthcare institutions currently face manpower shortages due to the COVID-19 pandemic. Therefore, the task of providing additional services to meet family members’ needs and relieve their stress was deferred by some healthcare institutions. The self-service system, known to be effective and efficient in other industries, was recommended for use in the healthcare industry. This study aims to explore an intensive care unit self-service system (ICU-SSS) designed for the family members of ICU patients. This study investigates the feasibility of the system by following a mixed method approach, including qualitative interviews and a quantitative survey. Firstly, interviews with five family members and five ICU staff members of a case hospital were conducted to identify the need to develop an ICU-SSS for the family member. Secondly, a survey was completed by 30 family members to evaluate the system. The interview results reveal nine categories of family members’ needs and the survey results show that the ICU family members assigned acceptable scores to all the ICU-SSS functions, except the importance of “Logistical information”. Based on these findings, the scientific and practical implications are discussed.

## 1. Introduction

Although open visitation has been highly recommended by critical care groups, more than 70% of Intensive Care Units (ICUs) in hospitals have restrictive visitation policies [1,2,3]. Because Asia is a collectivist rather than an individualistic society [4], Asian patients, in particular, rely on family members’ support. Meanwhile, religion plays an important role in the Asian population. Confucius’s teachings further emphasize the value of family and the obligation of each member of the family to support the others [5,6]. Therefore, family members play a key role when critical decisions regarding severely ill patients must be made [7], and their needs must also be considered and satisfied [8].

In Taiwan, family members can visit patients only during designated visiting hours, making physicians the primary source of information regarding a patient’s condition and progress. However, physicians usually have limited time in which they can provide the explanations. Consequently, family members may approach nursing personnel if they fail to understand physicians’ explanations. The ICU nurses were extremely busy ensuring that patients’ life-sustaining were running smoothly [9,10,11]. Meanwhile, most healthcare institutions are currently facing manpower shortages due to the COVID-19 pandemic. Therefore, it is often unfeasible for healthcare staff to provide additional services to meet family members’ needs and to relieve their stress. If they lack sufficient or complete information, family members may become anxious when making critical healthcare decisions for their loved ones, which may result in indirect tension in the medical staff–patient relationship and increased stress among all the concerned parties [12].

Khalaila summarized the research conducted worldwide over the past 30 years and indicates that family needs were still being neglected [13]. There are also different needs and assessments of satisfaction among family members [14,15]. Molter summarized 45 needs of ICU patients’ family members [16]. These items were then developed and incorporated into the Critical Care Family Needs Inventory (CCFNI), and Leske [17] divided them further into five primary categories, including the needs for support, comfort, information, proximity, and assurance, to reflect the multi-dimensional nature of the needs of the families of ICU patients. The CCFNI was used widely in various studies and proved to be sufficient [18,19].

Self-service technology (SST) allows users to access services without the participation of staff members [20]. SST is not subject to time and location constraints, is easy to use, avoids the need for additional service personnel, and saves time and money for both service users and providers. SST includes automatic teller machines, self-service gas stations, restaurants, and is widely used. Personal characteristics [21] and convenience, privacy, accuracy, and the versatility of the SST [22], can affect one’s willingness to use SST. Although SST can improve the efficiency of delivered services, reduce costs, increase competitiveness, enlarge market share, boost the level of customer satisfaction and increase royalties [23,24], SST-related applications are rarely used in the healthcare industry due to the fact that the digitization of the healthcare industry is slower than that in other industries [25].

Therefore, this study aims to explore an intensive care unit self-service system (ICU-SSS) designed for patients’ family members in a case hospital, and to evaluate the system to identify areas for further improvement.

## 2. Methods

**Figure 1:**
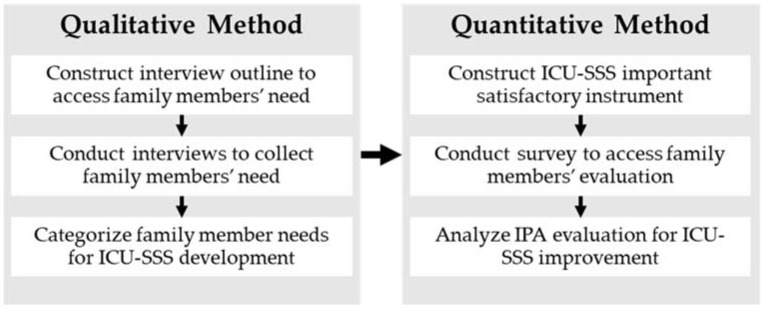
The research process.

This study investigated the feasibility of the ICU-SSS using a mixed method approach: first, interview the family members of a case hospital to identify their needs for developing the ICU-SSS, and, second, conduct a survey to evaluate the system. An outline of the research process is shown in Figure 1. Ethical approval for the study was obtained from the Institutional Review Board from the case hospital, which was one of the earliest facilities in Taiwan to utilize electronic medical records and demonstrated a high capacity for establishing an SSS. A team was formed to develop the ICU-SSS project. The development of the ICU-SSS system followed a prototyping life-cycle approach [26].

### 2.1. Qualitative Method: Interview and Identify Family Members’ Needs

Firstly, we obtained permission to use the Critical Care Family Needs Inventory (CCFNI) and combined the dimensions of dissatisfied family members, as indicated by Al-Mutair et al. [27], to develop a semi-structured instrument. Then, we conducted interviews with five family members of ICU patients and five ICU staff members with years of ICU work experience. The 5 ICU staff members contacted the family members almost every day and knew them well enough to provide thorough information about the family members’ needs. The interviews were conducted before and after the designated visiting hours using a semi-structured instrument (see Table A1) at the waiting area. The interviewer started with the question, “what are the common problems you bump into while visiting ICU patients?”, and then proceeded to ask questions on the semi-structured instrument. The length of the interview was kept within 10 min. During the interviews, the researcher would explain the purpose and related information of the study, starting with the structure of questions, checking the unmentioned items on the instrument with the participants, and finishing with an open-ended question. The interviews of ICU staff members were conducted before ward meeting using the same instrument, but started with the question, “what are the common problems that family members bump into while they visit ICU patients?”, to provide more information on the family members’ needs. The interviewed family members were mostly patients’ parents and self-reported to have an excellent interaction with the patients. The interviewed staff members all had ICU work experience above 6.5 years. The basic information of the interviewees is shown in Table A2. The response of the interviews was recorded and then processed for the ICU-SSS system design. The qualitative data analysis was performed by two researchers with ICU work experience and a background in management of information systems. They firstly transcribed the response of the interview and then classified this into categories. If a disagreement occurred, a system developer would join in and resolve the problem from a system development perspective.

The interview results reveal the family members’ needs, which were classified into nine categories: (1) ICU-related information, (2) patient information queries, (3) patient care information, (4) medical and health information, (5) palliative care, (6) consultations, (7) frequently asked questions (FAQs), (8) logistical information on the surrounding area, and (9) user input, suggestions, and comments. Table 1 shows the categories of ICU-SSS requirements and the relationship with CCFNI themes.

Referring to the principle of SST design proposed by Maguire [28], the information technology staff chose light blue and green as the primary colors of the webpages, and included simple graphics and instructions in the system to make users feel comfortable and relaxed. To encourage use and to display the readily available services to family members, the ICU-SSS was installed on a touch-screen panel outside the ICU. Family members had to input their provided ID numbers and passwords to log into the system. To ensure the security of patient data, once the patient was discharged from the ICU, the passwords became invalid. During the patient’s stay in the ICU, family members could operate the ICU-SSS and leave messages using the recording function, which was particularly beneficial for elderly family members who may have had difficulties with typing.

The main menu contains nine categories and is further broken down into two layers of screen design for users to drill down and search for the needed information. Two screen shots of the ICU-SSS are shown below. After logging into ICU-SSS, the homepage displays ICU information with care team information, patient information, online consulting information, and the tubes commonly used in patient care.

**Figure 2:**
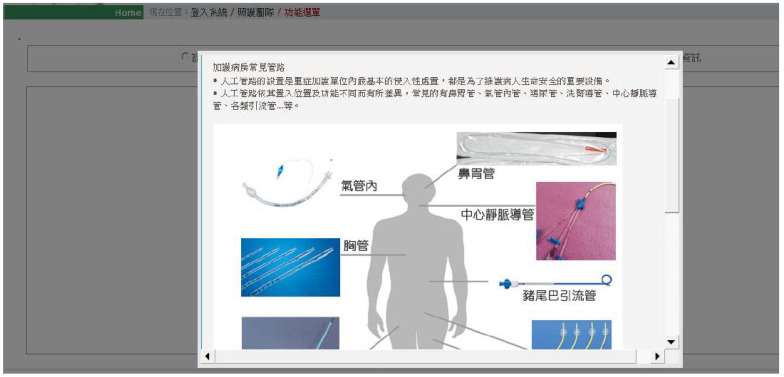
ICU-related information: introduction of commonly used tubes. Note: The ICU-SSS showed the relevant description of commonly tubes for patients’ care, started from upper right hand are nasogastric tube, central venous catheter, Pig-tail drainage tube, chest tube, intratracheal tube.

Photographs of the various tubes commonly used in patient care helped family members recognize and understand the patient treatments (Figure 2).

### 2.2. Quantitative Method: Survey and Evaluate the ICU-SSS

To check whether the system functions of ICU-SSS meet the users’ requirements, we adopted an importance–satisfaction instrument and conducted a survey to identify the priority of system improvement. The importance–satisfaction instrument combines measures of the users’ perceived performance and the importance of attributes, and classifies these attributes into four quadrants of a two-dimensional plot for further setting the priorities in allocating limited resources as the Importance–Performance Analysis (IPA) [20]. An expert panel with three scholars with a background in medical information systems was invited to ensure the content validity of the instrument. There are three parts of the questionnaire and the first two parts are the instrument of importance–satisfaction (shown in Table A3). The first part, comprising 9 questions, assessed the importance of the ICU-SSS functions on a 5-point Likert scale ranging from 5 (extremely important) to 1 (extremely unimportant). The second part, comprising 9 questions, determined the extent to which the ICU-SSS functions satisfied family members’ needs on a 5-point Likert scale, ranging from 5 (strongly agree) to 1 (strongly disagree). The third part included 12 questions that assessed participants’ basic data and a final item, the Acute Physiology and Chronic Health Evaluation (APACHE) score, which indicated the severity of the patient’s illness and was provided by the ICU nurses. Open-ended questions were designed to obtain suggestions regarding potential improvements to the ICU-SSS.

After the ICU-SSS was formally stable, we conducted the questionnaire survey at the ICU waiting area. In total, 37 family members were invited to use the system and fill the importance–satisfaction instrument. Thirty individuals completed the questionnaires, while the other seven declined the invitation and most of them were elderly family members. The quantitative data analysis, including the reliability of the instrument and descriptive statistics of IPA, was conducted by using SPSS 22.0 statistical software (IBM, Armonk, NY, USA). Each dimension is greater than 0.7 with a stronger correlation between the test questions and potential variables [29], see Table A4. To validate the questionnaire results, we conducted an informal follow-up interview with the head nurse of the ICU and with a family member who had experience caring for critically ill relatives before and after the implementation of the ICU-SSS.

## 3. Survey Results

### 3.1. Basic Information of the Participants

The participants comprised an equal number of men and women, the majority of whom were aged between 30 and 49 years, married, had a religious affiliation, spent 30 min or less traveling to the hospital, possessed a bachelor’s degree, often used other SST, were visiting parents, were the primary caregivers of a patient and had a strong emotional connection with the patient. Most patients remained in the ICU for 0 to 5 days and exhibited APACHE scores between 10 and 20 points. Table 2 reveals the detailed information on the survey participants.

### 3.2. Important Performance Analysis of ICU-SSS Functions

An IPA [30] was used to assess the extent to which the proposed functions satisfied the requirements of the family members of ICU patients and the importance of the function. Since IPA is used to classify system functions to indicate priorities in allocating limited resources for future improvements, the average scores of the importance–satisfaction from all users were calculated and plotted in the diagram. The average scores for the importance and performance of each ICU-SSS function are shown in Table 3.

**Figure 3:**
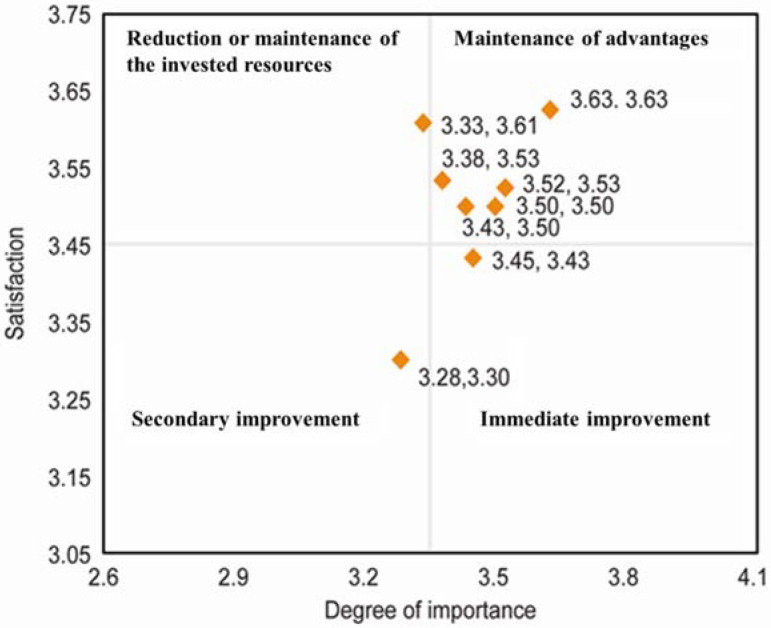
Importance–performance analysis matrix ICU-SSS functions.

The average scores for the degree of importance and the related satisfaction level of the functions as assessed by the family members were 3.35 and 3.45, respectively. In other words, participants agreed on the importance and performance of the ICU-SSS functions. Figure 3 presents an IPA matrix based on the average degree of importance and satisfaction yielded by the various functions. Most of the functions fall in the “Maintenance of advantages” quadrant, which means that survey participants are satisfied with these important functions. There is one function for the each of the other three quadrants.

According to the IPA analysis, the improvement strategies for these functions are listed in Table 4. For example, while the most important function was providing the “relevant medical and health information”, the respondents rated their level of satisfaction with this function as low. Accordingly, this function is among those that are of a high priority with respect to the areas for improvement.

A follow-up interviewing of a 40-year-old male family member revealed that he was satisfied with the information provided by the system and presented him with a sense of control. He further suggested installing the ICU-SSS touch-screen panel in a more secure area to avoid privacy problems when viewing patient information. The head nurse was also quite satisfied with the system, stating that it reduced the amount of time ICU nurses devoted to answering family members’ questions and thereby allowed them to concentrate on patient care.

## 4. Discussion

This study aimed to interview ICU patients’ family members in a case hospital to develop an ICU-SSS and survey their experience to evaluate the system to identify areas for further improvement. The interview results were developed into the ICU-SSS functions that met CCFNI’s three needs of support, information, and assurance. The need of comfort was achieved by a screen design and the proximity need was barely touched as a result of recording the family members’ voice messages and playing them to the patients for this version of the ICU-SSS. With the addition of more state-of-the-art techniques, such as virtual reality and augmented reality, the proximity category can be developed as a system function in later versions.

The survey results show that the most important and satisfied functions were providing “ICU-related information”, “Patient-related information”, “Relevant patient care information”, “Relevant consultation information”, and “Common Q&As”. The results confirm that the assurance and information dimension in the CCFNI was the most important need for the family members [31,32,33]. Regarding the feasibility of the system, the overall high satisfaction of the SST-ICU confirmed the results of prior studies that meeting family members’ needs can help them reduce their anxiety [33] and improve their satisfaction [33,34,35]. However, the function, “relevant medical and health information”, with high importance and relatively low satisfaction, requires an immediate improvement by extending the contents of relevant medical and health information to increase the users’ satisfaction. The results confirm those determined in a prior study [17] that if healthcare providers provided insufficient information regarding patient diseases and treatment, this would fail to satisfy family members’ informational requirements. Liang et al. [36] found that the high satisfaction and usage of the system can help to improve the doctor–patient relationship to enhance communication and the assessment of health information, and share the decision making. Due to ICU patients’ severe illness, family members played the key role of making critical decisions for them. A high satisfaction of using the SST-ICU can complete the doctor–patient family member relationship and smooth the decision making process. Most patients had APACHE scores of 10 to 20, which indicate a mild level of severity, and the family members expected a positive prognosis for them; thus, “palliative information” was unlikely to be needed either. For future studies, the correlation of patient satisfaction with the APACHE score or the length of stay in the ICU may be explored to reveal the effectiveness of the SSS-ICU system.

During the survey stage, we found that the majority of the family members of ICU patients were confident and proactively learned the system. They suggested expanding the ICU-SSS for use throughout the hospital to further provide customized services, enhance the quality of healthcare, and facilitate holistic care. Meanwhile, the elderly family members were hesitant to use the system and required more training time, confirmed by the research results of [20,21], which can be overcome by a more user-friendly screen design and customized training sessions.

## 5. Conclusions

Due to the patient’s limited ability to communicate, family members are an integral part of the treatment process and the patient’s safety in the ICU. All family members need sufficient and complete information regarding the ICU patients to make critical healthcare decisions for their loved ones. Since the family’s presence in the ICU may accelerate the patient’s recovery process [37], ICU staff can facilitate services centered on family members, ICU-SSS, and take advantages of their presence to support ICU patients.

The early prototypes aim to design, implement, and test an initial and usually highly simplified version of the system [38]. Our development of ICU-SSS followed the prototyping life-cycle approach of Naumann and Jenkins [39] and allowed users to evaluate the quickly established low-cost test system with an interactive process to discover their real needs. Meanwhile, we used the CCFNI, with good reliability and validity, as an interview base and invited an expert panel to review the instrument to evaluate the ICU-SSS. The validating process is rarely seen in other development systems, which sheds light on similar development systems and contributes to accelerate the process of achieving a satisfactory operational system. However, these results must be applied with caution, since the Asian population is culturally different from the Western population. Meanwhile, two possible biases were that the interview was carried out during the patient’s treatment phase and the researcher who conducted the interview was from the team that activated and explained the ICU-SSS. 

## Figures and Tables

**Table 1:** Functions of the ICU-SSS.

CCFNI	Main Menu	1st Layer	2nd Layer
Information	(1) ICU Information	ICU introduction	
		ICU visiting time and reception notice	
		Healthcare team	Physicians/nurses/pharmacist/RT/social workers
		ICU facilities	
		Common tubes	
Information	(2) Patient information	Glucose values/vital signs/medications/testing reports	
Information	(3) Patient care information	What can I do while visiting the patient?	Assistance with limb movement, relaxation skills, psychological support services
		Possible wards after leaving the ICU	Introduction to general wards, RCCs, RCWs, retirement centers
		How to care for the patient after he/she leaves the ICU	Nasal-gastric tube feeding, help cough up phlegm, bed bathing catheter, wound care
Information	(4) Medical and health information	Various diseases	Diseases classified by general internal medicine, cardiology, general surgery, neurosurgery, cardiac surgery, obstetrics and gynecology
		Common health examinations	Radiology, ultrasound, endoscopy, cardiac catheterization
		Common surgeries	general/neuro/cardiac surgery
Information	(5) Palliative care	Hospice care/hospice ward	Organ donation pain control, death symptoms/care
Information support	(6) Consultation information	Application of social welfare	Assist facility, foreign language services
		Certification	Diagnosis/death certificate, documents after patient’s death
		Helpline for relevant departments	Social work/funeral services, nutrition/drug/rehabilitation consultation
		Relevant support groups and websites	
Assurance	(7) FQAs		
Information	(8) Logistical/surrounding information	Dining/traffic/accommodation/floor map	
Assurance	(9) User input, suggestions, and comments		

Note: ICU: intensive care unit; RCCs: respiratory care centers; RCWs: respiratory care wards; FQAs: frequently asked questions.

**Table 2:** Basic information on the participants.

Characteristics	Class	Count (%)
Gender	Female	15 (50.0)
	Male	15 (50.0)
Age	20–29	7 (23.3)
	30–39	9 (30.0)
	40–49	9 (30.0)
	50–59	1 (3.3)
	60–69	4 (13.3)
Marriage	Married	17 (56.7)
	Not married	13 (43.3)
Education	Jr. High	6 (20.0)
	High school	4 (13.3)
	College	3 (10.0)
	University	14 (46.7)
	Graduate	3 (10.0)
Religion	No	9 (30.0)
	Yes	21 (70.0)
Home	<30 min	22 (73.3)
	30–60 min	8 (26.7)
Relationship with patient	Spouse	2 (6.7)
	Parent	17 (56.6)
	Offspring	3 (10.0)
	Brother/sister	3 (10.0)
	Other	5 (16.7)
Primary caregiver	Yes	16 (53.3)
	No	14 (46.7)
Interaction with patients	Excellent	21 (70.0)
	Good	8 (26.7)
	For duty	1 (3.3)
Length of stays in ICU	0–5	12 (40.0)
	6–10	5 (16.7)
	11–15	4 (13.3)
	16–20	3 (10.0)
	>20	6 (20.0)
Experience with self-services	Never	5 (16.7)
	Seldom	3 (10.0)
	Sometimes	7 (23.3)
	Often	15 (50.0)
APACHE	10–20	14 (46.7)
	21–30	7 (23.3)
	30–40	9 (30.0)

Note: APACHE: acute physiology and chronic health evaluation; ICU: intensive care unit.

**Table 3:** Importance–performance analysis of the ICU-SSS functions.

Functions	Importance	Performance	IPA Matrix
ICU-related information	3.38	3.53	(3.38, 3.53)
Patient-related information	3.63	3.63	(3.63, 3.63)
Patient care-related information	3.52	3.53	(3.52, 3.53)
Relevant medical and health information	3.45	3.43	(3.45, 3.43)
Palliative care	3.28	3.30	(3.28, 3.30)
Relevant consultation information	3.43	3.50	(3.43, 3.50)
Common questions and answers	3.50	3.50	(3.50, 3.50)
Logistical information for the surrounding area	2.61	3.07	(2.61, 3.07)
User input, suggestions, and comments	3.33	3.61	(3.33, 3.61)
Average	3.35	3.45	(3.35, 3.45)

**Table 4:** Opinions on the functions and related improvement strategies.

Quadrant	Functions of the Main Menu	Coping Strategies
**Relatively important, with a high degree of satisfaction** **(1st quadrant)**	ICU-related information Patient-related information Relevant patient care information Relevant consultation information Common questions and answers	Maintenance of advantages
**Important, with** **a low degree of satisfaction** **(2nd quadrant)**	Relevant medical and health information	Immediate improvement
**Less important, with a low degree of satisfaction (3rd quadrant)**	Palliative care; logistical information	Secondary improvement
**Not important, with a high degree of satisfaction** **(4th quadrant)**	User input, suggestions, and comments	Reduction in/maintenance of invested resources

Note: ICU: intensive care unit.

## Data Availability

The data of this study contain information that compromise the privacy of research participants and are not publicly available.

## Appendix A

**Table A1:** Structured question of the interview instrument.

Items	Check
ICU waiting room introduction	
ICU visiting time and reception notice	
Introduction of the healthcare team and relevant content	
Introduction to common ICU facilities	
Introduction to common patient tubes	
Patient’s glucose values, vital signs, wound progress, testing and inspection reports, medication	
What to do while visiting the patient	
Possible wards after leaving the ICU	
How to care for the patient after he/she leaves the ICU	
Information on various diseases	
Information on common health examinations	
Information on common surgeries	
Hospice care/hospice ward/pain control, issues of life continuation, death symptoms and care, article sharing	
Application of social welfare	
Relevant information for the certificate of diagnosis	

**Table A2:** Basic information regarding the interviewees.

Family Member	F-A	F-B	F-C	F-D	F-E
Gender	Male	Female	Male	Female	Female
Education	Bachelor	Bachelor	Jr. High	Bachelor	High school
Relationship to patient	Spouse	Parent	Parent	Parent	Parent
Interaction with patients	Excellent	Excellent	Good	Excellent	Excellent
Experience of self-service technology	None	Some	A lot	A lot	A lot
**Staff Member**	**S-A**	**S-B**	**S-C**	**S-D**	**S-E**
Gender	Male	Female	Male	Female	Female
Education	Bachelor	Bachelor	Bachelor	Bachelor	Bachelor
Position	Attending physicians	Nursing	Nursing	Nursing	Nursing
Work experience in ICU	12.4 years	6.5 years	10.3 years	6.5 years	9.1 years

**Table A3:** Instrument of IPA.

Functions	Importance					Performance				
	1	2	3	4	5	1	2	3	4	5
ICU-related information										
Patient-related information										
Patient care-related information										
Relevant medical and health information										
Palliative care										
Relevant consultation information										
Common Q&As										
Logistical information on the surrounding area										
User input, suggestions, and comments										
Average										

**Table A4:** Cronbach’s alpha of the ICU-SSS functions.

Functions	Cronbach’s Alpha
ICU-related information	0.753
Patient-related information	0.871
Patient care-related information	0.743
Relevant medical and health information	0.878
Palliative care	0.901
Relevant consultation information	0.790
Logistical information on the surrounding area	0.846
User input, suggestions, and comments	0.929
